# Extreme behavioural shifts by baboons exploiting risky, resource-rich, human-modified environments

**DOI:** 10.1038/s41598-017-14871-2

**Published:** 2017-11-08

**Authors:** Gaelle Fehlmann, M. Justin O’Riain, Catherine Kerr-Smith, Stephen Hailes, Adrian Luckman, Emily L. C. Shepard, Andrew J. King

**Affiliations:** 1 Department of Biosciences, College of Science, Swansea University, Singleton Park, Swansea, SA2 8PP UK; 2 Institute for Communities and Wildlife in Africa, Department of Biological Sciences, University of Cape Town, Rondebosch, 7701 South Africa; 3 Department of Computer Science, University College London, London, WC1E 6BT UK; 4 Department of Geography, College of Science, Swansea University, Singleton Park, Swansea, SA2 8PP UK; 5 Present Address: Department of Security and Crime, University College London, Gower Street, London, WC1E 6BT UK

## Abstract

A range of species exploit anthropogenic food resources in behaviour known as ‘raiding’. Such behavioural flexibility is considered a central component of a species’ ability to cope with human-induced environmental changes. Here, we study the behavioural processes by which raiding male chacma baboons (*Papio ursinus*) exploit the opportunities and mitigate the risks presented by raiding in the suburbs of Cape Town, South Africa. Ecological sampling and interviews conducted with ‘rangers’ (employed to manage the baboons’ space use) revealed that baboons are at risk of being herded out of urban spaces that contain high-energy anthropogenic food sources. Baboon-attached motion/GPS tracking collars showed that raiding male baboons spent almost all of their time at the urban edge, engaging in short, high-activity forays into the urban space. Moreover, activity levels were increased where the likelihood of deterrence by rangers was greater. Overall, these raiding baboons display a time-activity balance that is drastically altered in comparison to individuals living in more remote regions. We suggest our methods can be used to obtain precise estimates of management impact for this and other species in conflict with people.

## Introduction

Foraging strategies are intimately linked to the energy and time budgets of animal species^1^. For example, large herbivores such as elephants (*Loxondota africana*) spend their time using low-cost, low nutrient foraging strategies to meet their energetic requirements^2^, whilst cursorial predators such as cheetahs (*Acinonyx jubatus*) rely on short but intense bursts of energy to catch their high nutrient prey^3–5^. Between these extremes exists a diversity of foraging strategies that balance the investment in time and energy according to species and context^6^.

With unprecedented human-induced environmental change^7^, individuals may be able to modify their foraging behaviour (behavioural plasticity^8^) within their morphological and physiological constraints, and venture into human-modified environments to exploit high energy food resources^9^. Such ‘raiding’ behaviours can be particularly important for species with long generation times (because human-modified changes occur faster than evolutionary responses via natural selection^10^). Examples range from bears exploiting bins in North America^11^ to elephants consuming crops throughout large regions of Africa and Asia^12^, resulting in human-wildlife conflicts across the globe.

**Figure 1:**
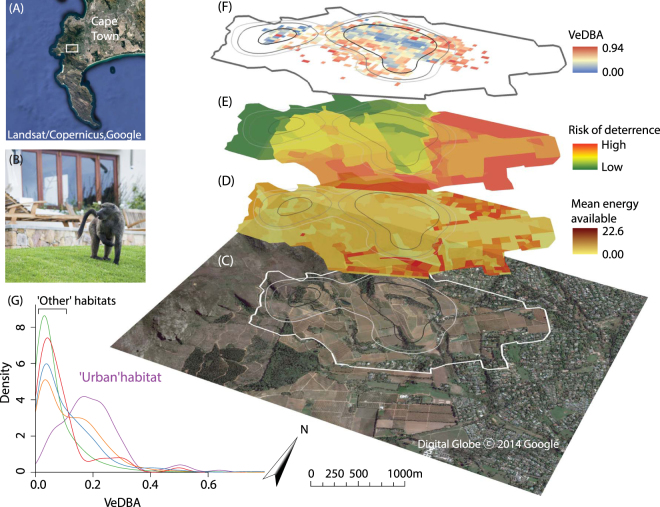
Estimation of energy available, risk of deterrence by ‘rangers’, and baboon activity levels in a human-changed landscape. (**A**) Study location in the Cape Peninsula, South Africa; (**B**) Raiding chacma baboon (*Papio ursinus*) in the study area; (**C**) Satellite image of the study area at the edge of Cape Town’s suburbs; (**D**) The energy available from potential food items, presented as the mean kcal/bite, per 150 m^2^ cell, ranging from light yellow for low energy (minimum = 0), to dark brown which is high energy (maximum = 22.6); (**E**) The risk of baboons being deterred by ‘rangers’ ranging from likely deterrence (red, score 22) to passive monitoring (green, score 0). (**F**) Activity of baboons represented as mean VeDBA (the Vectorial Dynamic Body Acceleration, in *g*) within 10 m^2^ cells. (**G**) The distribution density plot of the mean VeDBA score (in *g*) associated with GPS fixes (n = 6,273) coloured by habitats (fynbos in red, trees in green, meadows in blue, vineyards in orange and urban in purple). Note that for C–F the 95%, 90%, and 70% contour of the baboon troops’ home range is presented from light to dark grey respectively. Maps were created using ArcScene 10.4.1 (http://desktop.arcgis.com).

In the Cape Peninsula, South Africa (Fig. 1A), chacma baboons (*Papio ursinus*) not only raid farms, homes (Fig. 1B) and commercial properties in search of high-energy human derived foods, but also enter cars and even take food directly from people^13,14^. The severity of baboon raiding behaviour in the Cape Peninsula threatens human health and safety^15,16^ and poses significant risks to the baboons, with dozens of human-caused deaths each year^16^. In an attempt to prevent raiding behaviour, Cape Town’s city council employs a wildlife management company that uses baboon ‘rangers’ that ‘herd’ the baboons away from the urban edge using noise and paintball marker guns^17^. This management strategy reduces the time baboons spend in human-landscapes^16^, but urban and farm raiding - particularly by lone adult male baboons - still occurs^14^, resulting in troops staying within the vicinity of raiding spots^18^. We investigated the foraging strategies employed by raiding male baboons in Cape Town and estimate three key metrics: food resources available (representing energetic input), activity levels (representing energetic output), and management effort (representing risk). We focus on adult males – the most frequent raiders^19^ – in order to investigate the strategies by which these primates exploit human dominated landscapes and so expand their ecological niche^9,20^.

We hypothesised that flexibility in behaviour^8^ would be central to the baboons’ ability to cope with human-induced environmental changes and management strategies imposed upon them^17^, and tested a series of inter-connected predictions. First, we predicted that compared to ‘natural’ baboon habitats, urban spaces provide higher energy food rewards (prediction 1). Second, we expected that whilst urban spaces might provide greater rewards, baboons would experience increased risk of being deterred by field rangers^17^ (prediction 2), and thus engage in riskier and energetically costly activities in order to evade field rangers and navigate the complex urban topography (prediction 3). Consequently, if baboons are to exploit the opportunities presented by the urban space, we predicted they would employ behavioural strategies that are consistent with the baboons minimising costs and maximising rewards (prediction 4), resulting in extreme differences in behaviour when compared to (non-raiding) baboon populations (prediction 5).

## Methods

### Study subjects

One chacma baboon (*Papio ursinus*) troop with a long history of urban raiding^14^ was studied from 03/04/2014 to 21/07/2014 on the Cape Peninsula, South Africa (S -34.0349, E 18.4156). The focal troop comprised 10 adult and 3 sub-adult males, 20 adult and sub-adult females and approximately 30 juveniles. Our period of observation was relatively short but representative of a time of year when raiding was frequent in the population^21^ and when food availability in the natural environment was high^21^. We opportunistically collected data (*ad libitum*) on the occurrence of raids into urban spaces.

### Study area and baboon management

The study area was categorised as one of five broad habitat categories (fynbos, trees, meadows, vineyards and urban areas) based on researcher knowledge of the site and images from Google Earth (accessed 06/06/2014). Urban areas included private residential suburbs, vineyards and commercial properties (e.g., restaurants and wine producing plants). Fynbos was the indigenous vegetation within the Table Mountain National Park. The City of Cape Town and a local farm employed baboon ‘rangers’ to actively deter baboons from the vineyards and urban areas. Rangers monitored the troop, following their movements to keep them in sight, and used noise (shouts and whistles), physical presence and paintball guns to deter the baboons^17^. Two alternating teams of five field rangers managed the baboons on a daily basis from approximately 7am to 5 pm, with each team working four days on, four days off.

### Baboon tracking collars

Male baboons are known to be the most frequent raiders^19,22^, and 8 adult males within our focal troop were cage-trapped following the Baboon Technical Team’s approved protocol^23^ before being sedated by a certified veterinarian and fitted with a custom-built tracking collar (see supplemental information). Collars weighed less than 2.5% of the body mass of the baboons and were approved for use by Swansea University Ethics Committee (Swansea University IP-1314-5). Two collars failed to record data, decreasing the sample size to 6 individuals (see supplemental information for details of collar failure and subsequent improvements to this collar design).

Collars were equipped with sensors recording GPS every 5 minutes and acceleration in 3 axes at 40 Hz. Acceleration was recorded continuously at 40 Hz for 5 individuals and at 20 Hz for one individual (M2). GPS data were recorded from 7:30 am to 6:00 pm for a mean ± Standard Error (SE) of 10.3 ± 3.0 days, and a mean ± SE of 102 ± 5 GPS fixes per day, for each collared baboon. This resulted in 7,572 GPS fixes in total from which 7,428 fixes were associated with activity (acceleration) data. Any GPS fixes recorded before sunrise or after sunset according to the South African Astronomical Observatory were excluded, resulting in 6,325 fixes. Any pair of successive fixes more than 1 km apart were assumed to be errors, and removed, resulting in 6,274 fixes. The GPS receiver calculates standalone horizontal position to a quoted accuracy of 2.5 m, though, in practice, this depends upon satellites available and how the collar was positioned on the baboon at any time point, as well as the immediate environment surrounding the collared individual. Ad-hoc checks of the data where baboons crossed known landmarks indicate positional accuracy of < 10 m for our GPS data. GPS fixes were converted to Universal Transverse Mercator coordinate system before use in spatial analyses (below). Supplemental Table S1 summarises data collected by each male’s collar.

### Risks and returns

A map of the study region was divided into 150 m^2^ grid cells (n = 200). Due to the presence of a water dam, we removed one 150 m^2^ cell mostly covered by this feature, bringing the total number of cells to 199. We then estimated the risk of deterrence by rangers and food rewards for each cell.

Risk of deterrence by rangers was estimated by interviews with rangers (n = 11) employed to manage the troop. Interviews were carried out by GF and CK-S following the completion of the baboon data collection (see below) so as to not influence these data. Interviews were anonymous and conducted with the consent of the rangers and their employers. Rangers were provided with a map of the study area (Supplemental Fig. S1) and asked to colour in areas where, in their opinion, the baboons were allowed to be: at any time (green; score 0), some of the time over the course of a day (orange; score 1), or never, being consistently chased away (red; score 2). This provided us with 11 different maps and we used these data to derive a composite map created by summing all field rangers’ scores within each of the 150 m^2^ grid cells to provide an indication of the likelihood of deterrence. These data have previously been presented by Fehlmann *et al*.^24^. Note that using summed scores does not necessarily reflect ‘agreement’ between rangers (which can also impact on the troop’s space use), but agreement is high for urban environments nonetheless^24^. Further details of how the baboons respond to ranger management at a troop level can be found in Fehlmann *et al*.^24^.

To quantify potential energy returns, we surveyed baboons’ diets during focal observations of adult males (30 minute direct focal observations, see ‘Baboon activity’ below). For each food item, we defined the species (for vegetation), the part consumed, and estimated the volume of a baboon bite (a mouthful of this specific item; hereafter referred to as a baboon bite). In order to get an estimation of the weight of each food item consumed per baboon bite, we collected food material mimicking baboons’ foraging behaviour and weighed it. We then used data from the literature to estimate the energy content of one baboon bite based on its weight (Supplemental Table S2). When data on food energy content were not available, we estimated it by averaging the energy composition of similar food items (Supplemental Table S2). This gave us an estimated energy content for any given food item that was independent of absolute size, but meaningful with respect to a foraging baboon.

In order to map these potential energy returns, we surveyed the vegetation in 7.0 ± 3.5 random quadrats of 1 m^2^ (n = 1906 quadrats) for each of the 150 m^2^ grid cells. In each quadrat, we reported the presence and absence of each food type and estimated the number of baboon bites. We then averaged the energy per bite of all food items present in each quadrat to provide us with an estimation of the potential energy reward (in kcal) that a baboon would gain per bite of food in a given 150 m^2^ cell. For cells falling in urban habitats, we didn’t conduct analyses as quadrats but instead assessed the energy availability for each residence within a cell; recording presence or absence of food types. Whilst this approach provides a particularly coarse measure of available energy, we assume that it provides qualitatively accurate results for the broad scale habitat-level differences we investigate here.

### Baboon activity

To estimate baboon activity levels we used the Vector of the Dynamic Body Acceleration (VeDBA) calculated from acceleration data^25^. Raw acceleration data were first decomposed into static and dynamic components, using a running mean of two seconds to estimate the static acceleration^26^. Static acceleration provides information on the orientation of the device with respect to gravity, and the dynamic component reflects how much the animal is moving in each of the three dimensions (anterior-posterior, lateral and vertical). VeDBA is the square root of the sum of the squared dynamic acceleration values (measured in each of the three axes; x, y, z):

$$VeDBA=\sqrt{{X}^{2}+{Y}^{2}+{Z}^{2}}.$$

VeDBA therefore combines the dynamic part of the acceleration signal across three axes, providing a single measure of body motion and hence activity. We took the mean VeDBA around each GPS fix to be representative of the average level of activity at a given GPS location. A sensitivity test was conducted to assess the effect of changing the period over which acceleration data were averaged around GPS points. This showed a plateau at 60 s (Supplemental Fig. S2). We therefore computed the mean average VeDBA for 30 seconds before and after each GPS fix, and this information was used as an indication of the intensity of activity being performed by a collared baboon at any recorded time point.

To compare focal baboon activity budgets to those published in the literature, we conducted 30 minute direct focal observations of each male (n = 311; mean ± sd per baboon = 51.8 ± 0.7). At each minute we recorded the baboons’ instantaneous behaviour (classified as resting, grooming, foraging or travelling). Focal identity and times were randomly selected over the study period.

### Statistical tests

All statistical analyses were conducted in R version 3.1.1^27^ with α set at 0.05 and are reported in the supplementary “Source code”. Data are available from the Dryad Digital Repository at http://dx.doi.org/10.5061/dryad.10b93. To test for differences in energy availability and VeDBA according to habitat type (prediction 1), we conducted a Kruskal-Wallis test. To investigate relationships between the risk of deterrence by rangers and habitat type (prediction 2), we conducted Mantel tests whilst controlling for spatial autocorrelation (R package ‘vegan’^28^). We used a Kruskal Wallis test to see whether VeDBA (activity levels) changed according to habitat type and used a Linear Mixed Model, LMM (R package nlme^29^), to investigate if VeDBA changed as a function of risk of deterrence by rangers (prediction 3). For our LMM we log-transformed our response variable (VeDBA) to meet model assumptions and included management strategy score (0–22) as a fixed effect, whilst also including an exponential spatial correlation structure by including a nugget effect in the model. We fitted baboon identity as a random intercept to allow individuals to differ in their baseline VeDBA (this improved model fit according to AIC criterion (R package nlme^29^)). Homogeneity and homoscedasticity of residuals were checked to ensure model assumptions were met. To investigate whether baboons were minimising costs and maximising rewards (prediction 4), we calculated time spent in different habitat types and computed the distance of each GPS fix to the nearest urban edge (R package ‘spdep’^30,31^). To compare focal baboon activity budgets to those published in the literature (prediction 5), a Kruskal-Wallis test was used.

## Results

### Rewards

**Figure 2:**
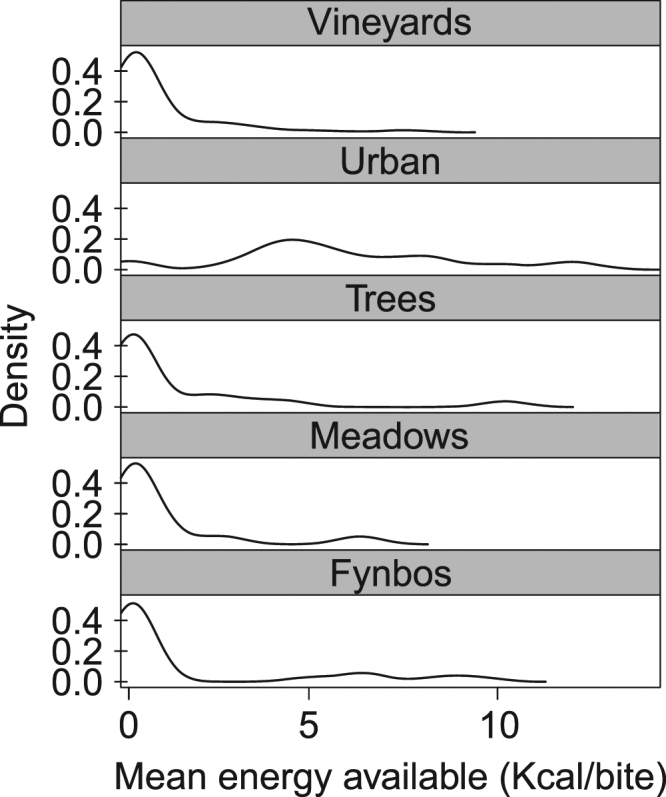
Estimation of energy availability in different habitats. Density plots showing the mean energy availability across 150 m^2^ cells (n = 199) found within each major habitat type.

Estimated food resource quality differed by habitat type (Kruskal Wallis: X^2^ = 51.7, df = 4, p < 0.001, n = 199, Fig. 1C,D), with 90% of the food resources sampled in the urban space yielding a potential energy intake ~10 times greater per bite than the natural environment (Fig. 2), in support of our first prediction.

### Risks

**Figure 3:**
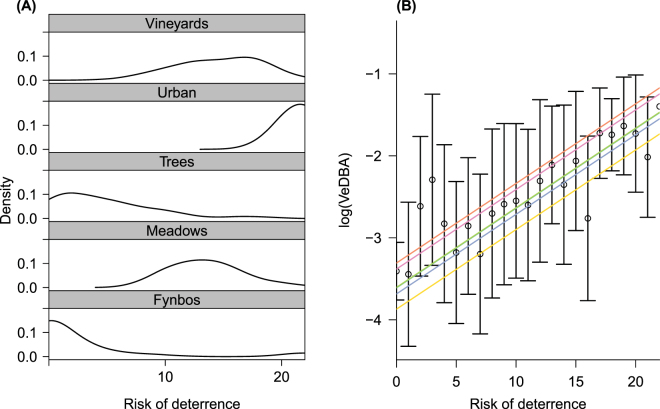
Rangers’ management strategy and its impact on baboons’ activity. (**A**) Density plot showing the baboon ranger management strategy across 150 m^2^ cells (n = 199) found within each major habitat type; (**B**) VeDBA (the Vectorial Dynamic Body Acceleration) in log scale, as a function of baboon ranger management strategy, with large risk of deterrence scores indicating baboons are actively deterred, and zero meaning baboons are passively monitored. Points and error bars represent mean ± SE for n = 6 males and lines represent individual responses estimated by a mixed model with a random intercept fitted for baboon identity, as indicated by different colours (only 4 colours are visible as there are two cases where individuals have the same intercepts).

Rangers were more likely to deter baboons in urban spaces (Partial Mantel test: management strategy vs. urbanisation, R = 0.66, p < 0.001, n = 199, Fig. 1C,E), which we assumed to translate to increased risk, supporting our second prediction. Baboons also showed significantly higher activity levels (VeDBA) in urban locations (Kruskal Wallis: VeDBA vs. habitat type, X^2^ = 576.34, df = 4, p < 0.001, n = 6274; Fig. 1F) and specifically where ranger management strategy was reported to be strictest (Linear Mixed Model, estimate = −0.077, t-value = −7.91, p-value < 0.001, n = 6274, Fig. 3). This contributed to VeDBA scores that were 4 times greater in urban habitats than in other habitats. Management effort also appeared to be 27 times greater in this environment (Fig. 1G), making urban spaces more costly for baboons to utilise than other habitats in their range (Fig. 1G), supporting our third prediction.

### Baboon raiding strategy

**Figure 4:**
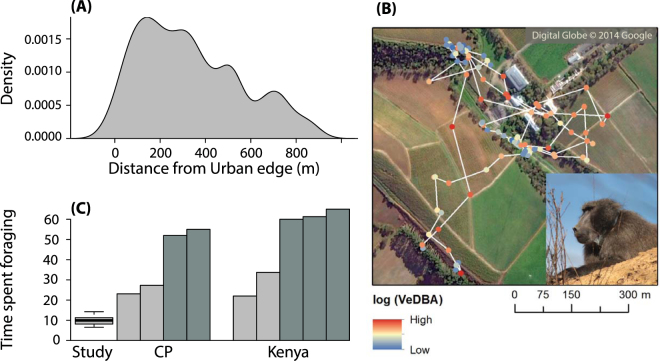
Raiding baboons’ sit-and-wait strategy and its consequence for foraging time budgets. (**A)** Baboon’s space use for collared males (n = 6) as the density of GPS locations found at a given distance from the urban edge. **(B)** An example of a GPS track for a baboon (inset, wearing collar) moving in and out of the urban habitat in a vineyard. GPS points represent 5-minute intervals from 7:30 to 18:00 (n = 113), with the colour of the points representing the mean VeDBA (the Vectorial Dynamic Body Acceleration) in log scale, from blue for low level of activity (minimum 0.01 *g*) to red for high level of activities (maximum 0.38 *g*), 30 seconds before and after the GPS location. White lines link GPS fixes for illustration. The map was created using ArcScene 10.4.1 (http://desktop.arcgis.com) **(C)** Time budget data for collared males in (n = 6), in comparison to other populations. Raiding populations are shown in light grey and non-raiding populations are in dark grey. Data for the Cape Peninsula (CP) from Hoffman^23^ (recorded in 2006 in winter) and data from Kenya from Altmann and Muruthi^32^ (recorded between 1984 and 1985 in dry season) and Strum^19^ (recorded between 1981 and 1984 in both dry and wet seasons).

We recorded 105 raids in 109 days during the study period (Supplemental Table 3) and identified a further 49 raids from collar data (between 5 and 17 urban incursions per baboon) over 35 days (60 days of GPS recordings across all males). Despite the high number of raids, the collared baboons spent just 1.8 ± 0.7% (mean ± SE; range: 0.5 to 5.2%) of their time in the urban space, but stayed in close vicinity (median [1^st^ and 3^rd^ quartile]; 300 m [149 m, 501 m] to urban space, Fig. 4A), mainly engaging in low-activity behaviours (mean ±  sd VeDBA = 0.08 ±  0.09) before engaging in brief (mean ± SE = 11.0 ± 1.5 minutes), high-activity (mean ±  sd VeDBA = 0.19 ±  0.11) forays into the urban space to raid (Fig. 4B).

The collared males spent just 9.5% ± 0.8% of their overall time budget foraging (Fig. 4C). This was significantly less than baboon troops elsewhere in Africa (prediction 5), which spend between 20.3% and 59.3% of their time feeding^13,19,32^ (Kruskal-Wallis rank test, X^2^ = 13.5, p < 0.001, Fig. 2C).

## Discussion

Throughout baboon species’ distribution, raiding individuals have shorter daily path lengths^18,33^, smaller home ranges^18^, invest less time in foraging^19^, and have greater reproductive success^19^ than non-raiding baboons. The apparent success of this strategy suggests there are substantial benefits to raiding despite significant associated risks^16,34,35^. However, no study has yet examined the behavioural processes by which raiding baboons (or any other raiding animal) exploit the opportunities and mitigate the risks to survive and thrive in a human-changed environment. Our study of raiding male baboons in Cape Town, which are subjected to intense management efforts to reduce conflict with people, has provided an estimation of the risks and rewards associated with baboon raiding.

We found that urban spaces afford access to high energy food resources, but these resources occur in areas where field rangers are likely to deter baboons. We expect that this threat of deterrence, in conjunction with complex urban topography, contributes to the higher baboon activity levels recorded in urban spaces when compared to other habitats in their range. Given that there is also potential for injury or death as a result of human-baboon conflicts^15,16^ (one study animal was killed by a dog while raiding during the study period), we suggest that the risks associated with raiding are high.

We have previously shown that the area bordering the urban space is (i) where inter-individual variation in field ranger management strategy is highest (i.e. sometimes they deter, sometimes they do not) and (ii) close to refuges^24^. Thus, baboons stay at the urban edge, engaging in low-activity behaviours near refuges, where risks of herding are variable^24^, and then engage in brief, high-activity forays into the urban space to raid when the opportunities arise. This last point is important; whilst baboons are more likely to be herded away from certain areas within their range, inconsistency in ranger responses^24^ and the urban topography mean that “once in a while” baboons get through, or are let through, to the urban space to raid (spending 1.8% of their time in urban space). Getting into the urban space to raid, even infrequently, is incentive enough to persist with raiding attempts: the baboons spend almost all of their time close to the urban space (the majority of GPS points are located within 500 m of the urban edge) and take opportunities to exploit urban resources, with 49 raids in 35 days.

Based on our findings, the management strategies in the study period appeared to be contributing to increased costs of moving through urban spaces. However, baboon activity levels in urban spaces, whilst high, were not maximal, as VeDBA values recorded for running were 1.6 times greater than the maximum VeDBA recorded for urban areas (Supplemental Fig. S3). Furthermore, even if ranger effort to deter baboons was theoretically 27 times higher in the urban areas (Fig. 3), this only translated to a four-fold increase in the raiding costs experienced by baboons. Whilst this may have been due to an exaggeration of real effort by baboon field rangers during the interviews, it is likely that rangers could not increase their effort in urban areas sufficiently, since baboons were not operating at maximum activity when raiding.

Future research and management decisions should consider complementary or additive approaches to keep the baboons out of urban areas. Bins were the most raided item/location during this study (Supplemental Table S3) and so more time and resources could be devoted to ensuring these are managed more effectively, as suggested by Kaplan^36^. Improved waste management is frequently used in countries where human-wildlife conflicts exist with particularly dangerous species such as bears^37,38^ and has proven important in mitigating conflict in urban areas^39^. There is also potential for decreasing baboon raiding efficiency through increasing the travel time to urban areas^40^. This could be achieved by either building baboon proof fences in judicious places, which would require the baboon to circumnavigate the obstacle, or by implementing a no-go buffer zone around the urban edge, preventing baboons from adopting a sit, wait, and observe strategy^24^. Similarly, technological development of ‘virtual fences’ where sounds are played via loud-speakers when baboons approach particular locations, currently being tested in Cape Town^41^, may provide a novel way of introducing such a buffer zone, but the impacts on other wildlife and reduction in access to natural land for baboons need to be determined first^42,43^.

Given that our sample is restricted to adult males which are habitual raiders, it would be worthwhile investigating the effect of management strategies according to baboon age-sex classes or even personality types^10,44,45^. This may help explain some of the underlying mechanisms linked to the consistent inter-individual differences in raiding propensities according to sex^19,22,46^, or even between individuals of the same sex^22^, for a variety of species in conflict with humans. This approach could be particularly important since association networks are known to influence crop-raiding behavior in male African elephants^22^, and high ranking adult male baboons (but not low ranked males) can influence the foraging decisions and space use of the entire group^47–49^. Therefore, where certain individuals have a disproportionate influence within their social units (and play “keystone” roles^50^), it could be more efficient to attempt to manage these individuals.

In summary, we have used data acquired from raiding male baboons fitted with high-resolution (GPS and motion sensor) tracking collars to show that exploitation of the human-modified environment is costly. We found baboons engaged in brief, high-activity forays into the urban space, where high energy resources are located. Consequently, the raiding male baboons in the Cape Peninsula exhibit a time-activity balance that is drastically altered in comparison to individuals in populations living in more remote regions^18,19,32^; switching from a time-expensive low-risk foraging strategy, to brief and risky high-activity forays into the urban space. Our results also present unequivocal evidence of an extreme behavioural flexibility. Such behavioural flexibility has long been considered a central component of a species ability to cope with human-induced environmental changes^10^, but explicit quantification of its occurrence and the associated trade-offs has been lacking in wild animal populations.

## Electronic supplementary material

Supplementary information
