# Supplementary material for: Extreme behavioural shifts by baboons exploiting risky, resource-rich, human-modified environments
Source: Sci Rep. 2017 Nov 8;7:15057. doi: 10.1038/s41598-017-14871-2 (PMC5678166; doi:10.1038/s41598-017-14871-2)
Supplement: Supplementary file 1 — Supplementary information [file 41598_2017_14871_MOESM1_ESM.pdf]

SUPPLEMENTAL INFORMATION

Extreme behavioural shifts by baboons exploiting risky, resource-rich, human-modified environments

Authors: Gaelle Fehlmann*[1], M. Justin O'Riain[2], Catherine Kerr-Smith[1]†, Stephen Hailes[3], Adrian Luckman[4], Emily L. C. Shepard[1], Andrew J. King[1]

Affiliations:

[1]Department of Biosciences, College of Science, Swansea University, Singleton Park, Swansea, SA2 8PP, UK.

[2]Institute for Communities and Wildlife in Africa, Department of Biological Sciences, University of Cape Town, Rondebosch, 7701, South Africa.

[3]Department of Computer Science, University College London, London, WC1E 6BT, UK.

[4]Department of Geography, College of Science, Swansea University, Singleton Park, Swansea, SA2 8PP, UK.

*Correspondence to: 798266@swansea.ac.uk

†Present: Department of Security and Crime, University College London, Gower Street, London WC1E 6BT.

Additional information (Methods: capture)

For five days, cages were baited with corn and placed within the core home range of the study troop so that the baboons readily entered and consumed the food (corn). On the day of trapping, once the baboon was settled and feeding, the door was shut by an observer pulling a cord that released it. The baboons were anaesthetized using low impact 1.5ml plastic dart deployed from a pole (Daninject or Telinject), with 0.6ml of Ketamine (2mg/kg). Once sedated, the collar was fitted to the baboon and adjusted accordingly. The collar was made sufficiently tight so that it may not be pulled over the baboons head, nor get caught in vegetation, but sufficiently loose as to be comfortable and not hinder the animal’s respiration or ingestion and rotate around its neck freely. The smooth inner surface of the collar minimised risk of rubbing. Once the collar had been fitted the baboon was administered with 0.6ml of Medetomidine (0.02 mg/kg) in order to reverse the effects of the sedation. The baboon was then placed back into the cage until it had regained full mobility and awareness. Once recovered, it was released from the cage and re-joined the troop.

Additional information (Methods: collar failure)

Whilst we completed robust testing in the lab for our custom-designed collars, we experienced a high rate of collar failure. Two of the collars did not record and several stopped working due to water damage or due to a failure in the memory cards (details provided in Table S1). These prototype collars were significantly enhanced after this study; learning from our mistakes we created a second version that we describe in Fehlmann et al.[S1]. If you are looking to replicate this work, or do something similar, please follow the full end-to-end process we describe in the supplementary material of Fehlmann et al.[S1] in order to avoid encountering the same issues we had during the study we present here.

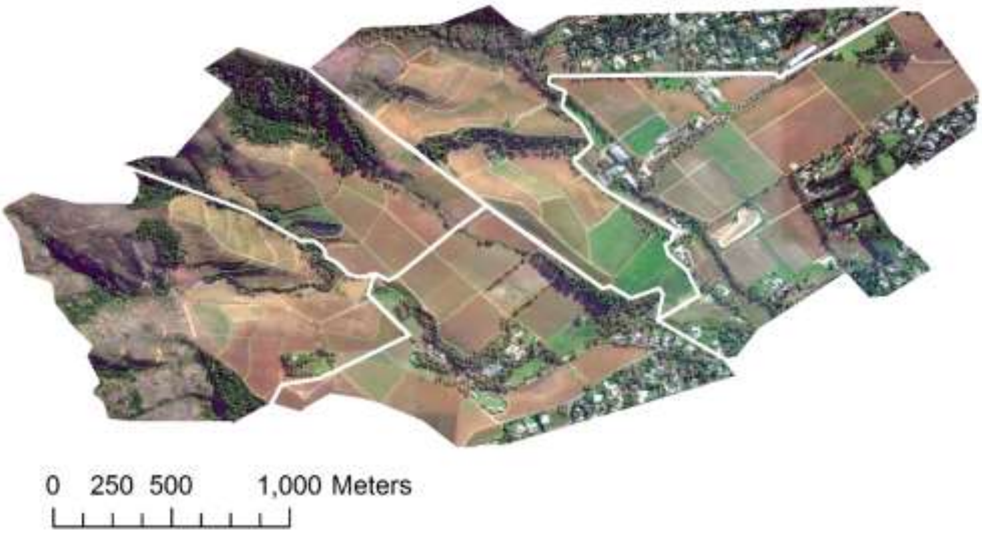

Fig. S1. Map of study area (Methods: Risk and returns).

Map of the area given to the rangers during the interviews. Rangers were asked to colour in areas between the white lines where, in their opinion, the baboons were allowed to be: at any time (green), some of the time (orange), or never (red). Areas were defined by specific landmarks such as farm boundaries, roads or tree lines. The satellite image is issued from Google Earth (access date: 6/6/2014).

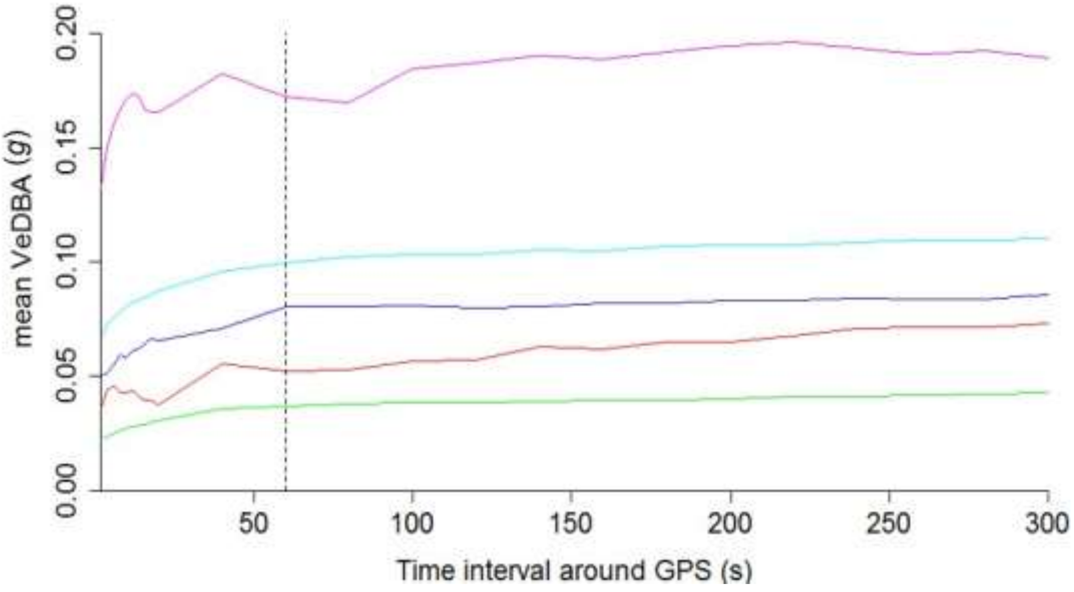

Fig. S2. Sensitivity analysis for VeDBA (Methods: Space use and activity).

Sensitivity analysis to select the time interval around a GPS fix (n=6,273) for the computation of the mean VeDBA (*g*). The sensitivity analysis has been carried in each different habitat type fynbos, trees, meadows, vineyards and urban (red, green, blue, cyan, magenta, respectively). The vertical line represents the time interval chosen.

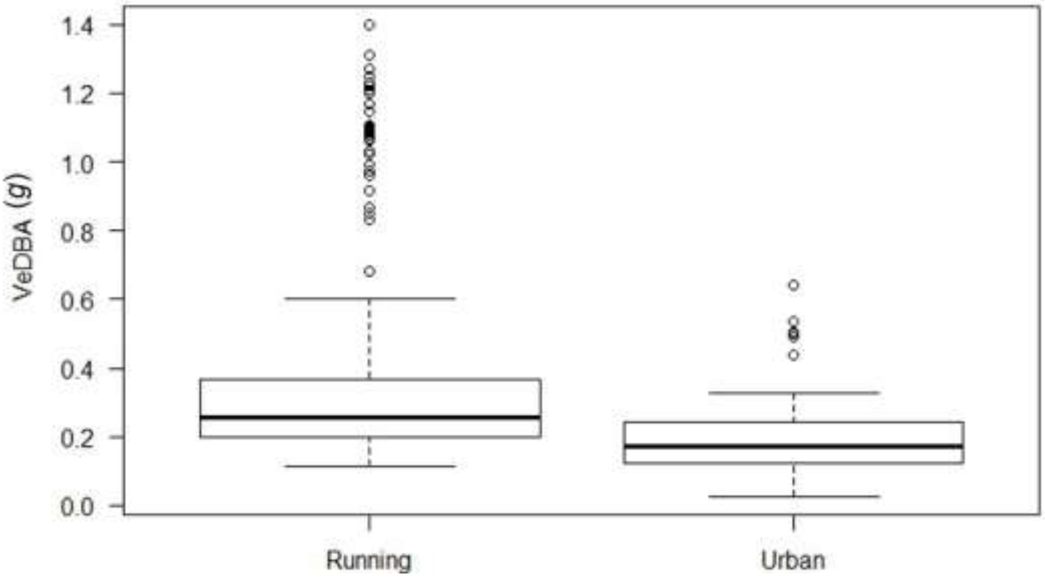

Fig. S3. VeDBA comparison (Discussion).
The Vectorial Dynamic Body Acceleration (VeDBA) measured while focal baboons were running (n = 169 events, defined by video footage: Fehlmann et al. 2017) and while the baboons were in the urban area (n = 116 events, defined by GPS fixes).

Table S1. Details of the recording period for each individual in days (Methods: Baboon tracking collars).

The collars were intended to record for 50+ days but stopped working due to water damage or due to a failure in the memory cards (details provided). These prototypes were significantly enhanced in a second version that we describe in Fehlmann et al.[S1]. Overall, collars recorded 7,573 GPS fixes, among which 7,428 were associated with activity data and 6,322 were recorded during daytime. From those, 6,274 GPS fixes met our accuracy criterion. The collars were removed after 3 months.

Individual	Start	End	Duration	Total GPS fixes	GPS fixes recorded during daytime	Reason for the interruption of data recording
M1	03/05/2014	15/05/2014	12	1,485	1,295	Memory card error
M2	21/05/2014	31/05/2014	10	1,473	1,183	Memory card error
M3	21/05/2014	06/06/2014	16	1,499	1,239	Memory card error
M4	30/05/2014	03/06/2014	4	602	501	Water damage
M5	31/05/2014	10/06/2014	10	1,391	1,152	Memory card error
M6	11/06/2014	19/06/2014	8	1,123	904	Damage to antenna

Table S2. List of all food items sampled (Methods: Risk and returns).
Details of baboon food items recorded during environmental sampling. Bite size (in g) was estimated based on behavioural focal observations. Sources for the nutritional characteristics of the different food items are given in square brackets and referenced at the end of this document.

Food Item	Energy	Bite size	Kcal/Bite	Size estimation	Comment
Acacia sp., seed [S2]	427.3	0.1	0.5	1 unit	given as *Acacia nicolota*, seed
Bin [S3]	NA	NA	10.0	NA	* estimated with vegetable/fruit peel and bread
Bread [S3]	266.0	8.8	23.4	1/5 slice	given as white bread, commercially prepared
Bromus diandrus, seed [S3]	352.0	0.1	0.2	1 unit	given as barley, pearled, raw
Bromus diandrus, stem [S3]	102.9	0.3	0.3	1 unit	given as barley, aerial part, fresh
Citrus lemonum, fruit [S3]	29.0	11.6	3.4	1/5 unit	given as lemon, raw, without peel
Egg [S3]	143.0	9.5	13.6	1/4 unit	given as whole, raw, fresh
Ficus sp., fruit [S3]	74.0	25.0	18.5	1/2 unit	given as fig, raw
Fruit [S3]	NA	NA	12.8	NA	* estimated with different fruits
Hypochoeris radica, leaf [S2]	NA	NA	0.4	3 leaves	* estimated with leaves
Hypochoeris radica, root [S3]	NA	NA	0.3	1/3 root	* estimated with radish
Lawn [S2]	NA	NA	0.4	NA	* estimated with small annuals
Lupinus sp., leaf [S2]	90.9	0.5	0.5	3 leaves	given as *Lupinus albus*, aerial part, fresh
Myrtillus sp., fruit	NA	NA	2.1	1 unit	* estimated with berries
Olea sp., fruit [S3]	145.0	3.4	4.9	1 unit	given as olive, bottled, green
Ornithopus compressus, aerial part [S2]	NA	NA	0.4	1 leaf	* estimated with leaves
Oxalis sp., flowers [S2]	NA	NA	0.4	1 unit	* estimated with leaves
Oxalis sp., leaf [S2]	NA	NA	0.4	5 leaves	* estimated with leaves
Pennisetum clandestinum, stem [S2]	87.5	0.3	0.3	1 unit	given as aerial part, fresh
Persea americano, fruit [S3]	160.0	13.6	21.8	1/10 unit	given as avocado, raw, all commercial variety
Pinus pinea, seed [S3]	673.0	0.2	1.1	1 unit	given as pine nut, dried
Protea tree, seed [S3]	NA	NA	0.8	1 unit	* estimated with seeds
Psidium guajava, fruit [S3]	68.0	11.0	7.5	1/5 unit	given as guava, raw
Quercus sp., seed [S3]	387.0	5.8	22.6	1 unit	given as acorn, raw
Rahpanus raphinastrum, flower [S2]	NA	NA	0.4	1 unit	* estimated with leaves
Rahpanus raphinastrum, leaf [S2]	NA	NA	0.4	3 leaves	* estimated with leaves
Rahpanus raphinastrum, root [S3]	NA	NA	0.3	1/3 root	* estimated with radish
Rosa sp., hip [S3]	162.0	3.8	6.2	1 unit	given as rose, hip
Triticum sp., seed [S2]	376.9	0.1	0.2	1 seed	given for grain
Triticum sp., stem [S2]	125.7	0.3	0.4	1 unit	given forage, fresh
Veg patch [S3]	NA	NA	4.0	NA	* estimated with vegetables
Vicia tetrasperma, aerial part	NA	NA	0.4	1 unit	* estimated with leaves
Vitis vinifera, fruit [S3]	69.0	5.0	3.5	1 berry	given as grape, red or green, raw
Watsonia sp., bulb [S3]	NA	NA	12.5	1/3 unit	* estimated with tuberous vegetables

Table S3: Details of the witnessed raids (Results: Baboon raiding strategy).
Data or raiding was opportunistically recorded during the study period (from the 03/04/2014 to the 21/07/2014, i.e. 109 days)**.**

Target	Number of raids
Bin	57
Restaurant	23
Garden	7
People	6
Car	4
Unattended Backpack	2
House	2
Not identified	4
Total	105

Supplemental References

S1. Fehlmann, G., O'Riain, M. J., Hopkins, P. W., O'Sullivan, J., Holton, M. D., Shepard, E. L. C., King, A. J. (2017) Identification of behaviours from accelerometer data in a wild social primate. *Animal Biotelemetry* 5:6.

S2. U.S. Department of Agriculture (2015) USDA National Nutrient Database for Standard Reference.

S3. Johnson, C. A., Raubenheimer, D., Rothman, J. M., Clarke, D. & Swedell, L. (2013) 30 days in the life: daily nutrient balancing in a wild chacma baboon. *PLoS One* 8, e70383.

Source code

This document provides all R codes used for all analyses of data presented in:

Extreme behavioural shifts by baboons exploiting risky, resource-rich, human-modified environments

To use these scripts requires the following R packages:

```
library(vegan)
library(dunn.test)
library(MASS)
library(nlme)
library(plyr)
```

<u>To test differences in energy availability and VeDBA according to habitat type (prediction 1)</u>

Loading the data

```
grid=read.csv("EnvironmentData.csv")
```

Kruskal Wallis Test

```
kruskal.test(FoodAvail~Habitat,data=grid)

##
##  Kruskal-Wallis rank sum test
##
## data:  FoodAvail by Habitat
## Kruskal-Wallis chi-squared = 51.698, df = 4, p-value = 1.595e-10
```

⇨ Food availability varies according to habitat type

```
dunn.test(grid$FoodAvail,grid$Habitat,method="bonferroni")

##   Kruskal-Wallis rank sum test
##
## data: x and group
## Kruskal-Wallis chi-squared = 51.698, df = 4, p-value = 0
##
```

```
                                Comparison of x by group
##                                       (Bonferroni)
## Col Mean-|
## Row Mean |     Fynbos    Meadows      Trees      Urban
## ---------+--------------------------------------------
##  Meadows |  -0.560504
##          |     1.0000
##          |
##    Trees |   0.005437   0.534968
##          |     1.0000     1.0000
##          |
##    Urban |   4.724985   4.454984   4.315772
##          |     0.0000     0.0000     0.0001
##          |
## Vineyard |  -0.562177   0.218405  -0.506213  -6.946080
##          |     1.0000     1.0000     1.0000     0.0000
```

To investigate relationships between the risk of deterrence by rangers and habitat type (prediction 2)

Generating distance matrices:

```
DistsRisk <- dist(grid$Risk)
DistsHabitat <- dist(grid$HabLevel)

Dists <- as.matrix(dist(cbind(grid$Lat,grid$Long)))
Dists.inv <- 1/Dists
diag(Dists.inv) <- 0
```

Computing the Mantel test with 10 000 permutations:

```
mantel.partial(DistsRisk,DistsHabitat,Dists.inv,
            method="spearman",permutations=10000)

##
## Partial Mantel statistic based on Spearman's rank correlation rho
##
## Call:
## mantel.partial(xdis = DistsRisk, ydis = DistsHabitat, zdis = Dists.inv,
method = "spearman", permutations = 10000)
##
## Mantel statistic r: 0.6598
##       Significance: 9.999e-05
##
## Upper quantiles of permutations (null model):
##    90%    95%  97.5%    99%
## 0.0271 0.0365 0.0451 0.0558
## Permutation: free
## Number of permutations: 10000
```

⇨ risk of deterrence by rangers and habitat type are highly correlated (controlling for spatial autocorrelation)

Kruskal Wallis test to see whether VeDBA (activity levels) changed according to habitat type (prediction 3)

Loading the data:

```
GPSi=read.csv("CollarData.csv")
```

VeDBA and Habitats:

```
kruskal.test(VeDBA~Habitat,data=GPSi)

##
##  Kruskal-Wallis rank sum test
##
## data:  VeDBA by Habitat
## Kruskal-Wallis chi-squared = 576.34, df = 4, p-value < 2.2e-16
```

⇨ VeDBA varies according to habitats

```
dunn.test(GPSi$VeDBA,GPSi$Habitat,method="bonferroni")

##   Kruskal-Wallis rank sum test
##
## data: x and group
## Kruskal-Wallis chi-squared = 576.3351, df = 4, p-value = 0
##
##
##                            Comparison of x by group
##                                  (Bonferroni)
## Col Mean-|
## Row Mean |     Fynbos    Meadows      Trees      Urban
## ---------+--------------------------------------------
##  Meadows |   1.514556
##          |     0.6494
##          |
##    Trees |  -1.991446  -12.03757
##          |     0.2322     0.0000
##          |
##    Urban |   6.308603   8.325330   13.82481
##          |     0.0000     0.0000     0.0000
##          |
## Vineyard |   2.228992   2.342222   21.06535  -7.736911
##          |     0.1291     0.0958     0.0000     0.0000
```

Linear Mixed Model to investigate if VeDBA changed as a function of risk of deterrence by rangers (prediction 3).

Using Boxcox method to identify best transformation:

```
box=boxcox(lm(VeDBA~Risk,data=GPSi),lambda = seq(-0.1,0,by=0.001))
```

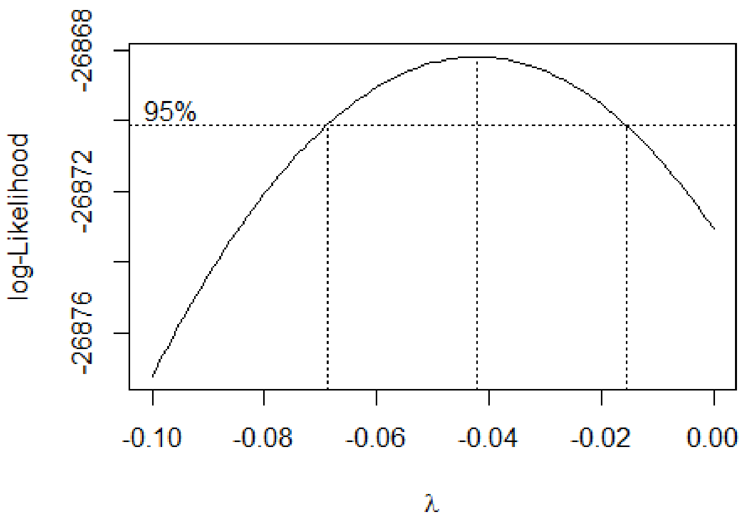


```
box[[1]][which(box$y==max(box$y))]

## [1] -0.042
```

⇨ Use Log transformation

```
GPSi$VeDBA.tr=log(GPSi$VeDBA)
```

Linear Mixed Model (LMM) with Individual as a random effect:

```
VOm1=lme(VeDBA.tr ~ Risk, random = ~ 1+Risk|ID, data=GPSi)
```

Investigating the need for a model taking into account spatial auto-correlation:

```
plot(Variogram(VOm1, form = ~ Long + Lat))
```

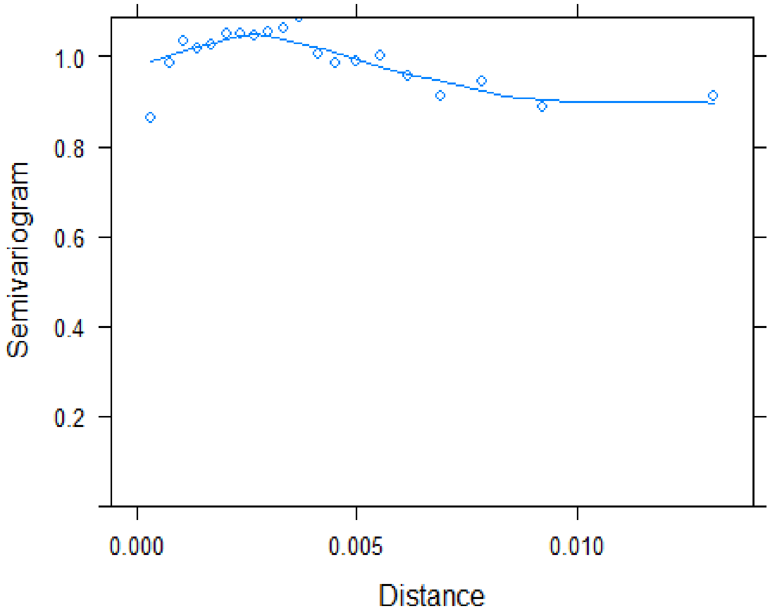

⇨ The presence of a slope indicates the effect of spatial auto-correlation in the data

Testing the different auto-correlation methods:

```
VOm1spcorr1a=update(VOm1, correlation=corExp( form = ~ jitter(Lat) + Long,
                  nugget = T))

VOm1spcorr2a=update(VOm1, correlation=corRatio( form = ~ jitter(Lat) + Long,
nugget = T))

VOm1spcorr3a=update(VOm1, correlation=corSpher( form = ~ jitter(Lat) + Long,
nugget = T))

VOm1spcorr4a=update(VOm1, correlation=corLin( form = ~ jitter(Lat) + Long,
                 nugget = T))

VOm1spcorr5a=update(VOm1, correlation=corGaus( form = ~ jitter(Lat) + Long,
                 nugget = T))
```

```
anova(VOm1spcorr1a,VOm1spcorr2a,VOm1spcorr3a,VOm1spcorr4a,VOm1spcorr5a,
        test=F)

##                Model df      AIC      BIC    logLik
## VOm1spcorr1a     1  8 15446.01 15499.96 -7715.007
## VOm1spcorr2a     2  8 16422.56 16476.51 -8203.281
## VOm1spcorr3a     3  8 16422.56 16476.51 -8203.281
```

```
## VOm1spcorr4a      4  8 16422.56 16476.51 -8203.281
## VOm1spcorr5a      5  8 16421.83 16475.78 -8202.916
```

⇨ Selection of the exponential correlation form

```
anova(VOm1,VOm1spcorr1a)

##                Model df      AIC      BIC    logLik   Test L.Ratio p-value
## VOm1               1  6 16418.56 16459.03 -8203.281
## VOm1spcorr1a       2  8 15446.01 15499.96 -7715.007 1 vs 2 976.549  <.0001
```

⇨ Selection of the model taking into account spatial-autocorrelation over the simple mixed model

Investigating individual effects as a random factor:

```
VOm1spcorr1a=lme(VeDBA.tr ~ Risk, random = ~ 1+Risk|ID, correlation=corExp(
form = ~ jitter(Lat) + Long, nugget = T), data=GPSi)

VOm1spcorr1b=lme(VeDBA.tr ~ Risk, random = ~ 1|ID, correlation=corExp( form =
~ jitter(Lat) + Long, nugget = T), data=GPSi) #best

VOm1spcorr1c=gls(VeDBA.tr ~ Risk, correlation=corExp( form = ~ jitter(Lat) +
Long, nugget = T), data=GPSi)



anova(VOm1spcorr1a,VOm1spcorr1b,VOm1spcorr1c)

##                Model df      AIC      BIC    logLik   Test   L.Ratio p-value
## VOm1spcorr1a       1  8 16422.46 16476.41 -8203.229
## VOm1spcorr1b       2  6 15441.05 15481.51 -7714.526 1 vs 2  977.4071  <.0001
## VOm1spcorr1c       3  5 16717.01 16750.72 -8353.502 2 vs 3 1277.9534  <.0001
```

Chosen model:

> *VOm1spcorr1b=lme(VeDBA.tr ~ Risk, random = ~ 1|Ind, correlation=corExp(form = ~ jitter(Lat) + Long, nugget = T), data=GPSi)*

Checking model assumptions:

```
VOm=VOm1spcorr1b
resid=resid(VOm)
pr=predict(VOm)
```

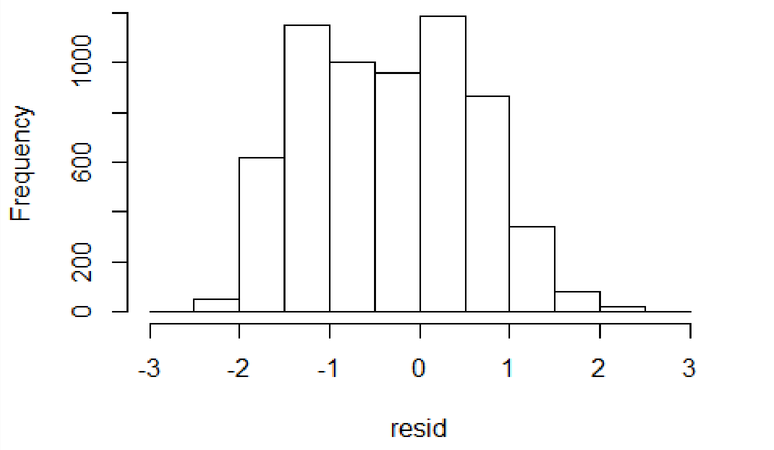

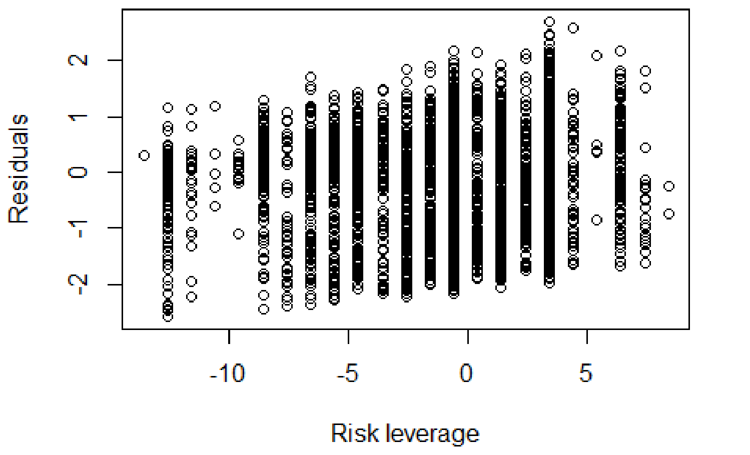

⇨ Model assumptions met.

<u>To compare focal baboon activity budgets to those published in the literature (prediction 5)</u>

Loading study data:

```
TBudg=read.csv("TimeBudget.csv",header=T)
percent=prop.table(as.matrix(TBudg[,2:5]),2)*100
colnames(percent)=c("Soc.p","Res.p", "Mov.p", "For.p")
TBudg=cbind(TBudg,percent)
TBudg=TBudg[TBudg$ID!="OR",] #Individual died during the study
```

Data from literature:

```
TBudgStrum=data.frame(Feeding=c(mean(33.68,28.33,24.31,25.86,24.12,38.12),
           mean(61.29,37.98,54.50,56.57,45.93,41.66)),ID=c("WBY","PHG"),
```

```r
            Strat=c("Raiders","Non Raiders"), Origin="Kenya")

TBudgAltmann=data.frame(Feeding=c(22,65,60),ID=c("LDG","HOK","ALT"),
            Strat=c("Raiders","Non Raiders","Non Raiders"), Origin="Kenya")

TBudgKenya=rbind(TBudgAltmann,TBudgStrum)
TBudgKenya$ID=as.character(TBudgKenya$ID)
TBudgKenya$Origin=as.character(TBudgKenya$Origin)
TBudgKenya$Strat=as.character(TBudgKenya$Strat)
TBudgKenya=TBudgKenya[order(TBudgKenya$Feeding),]

TBudgHoffman=data.frame(Feeding=c(27.3,55,23.1,52.0),
            ID=c("RH","BB","TK","KK"), Strat=c("Raiders","Non Raiders",
            "Raiders","Non Raiders"), Origin="Cape Peninsula")

TBudgHoffman$ID=as.character(TBudgHoffman$ID)
TBudgHoffman$Origin=as.character(TBudgHoffman$Origin)
TBudgHoffman$Strat=as.character(TBudgHoffman$Strat)
TBudgHoffman=TBudgHoffman[order(TBudgHoffman$Feeding),]

AllData=data.frame(Feeding=c(TBudg$For.p,TBudgHoffman$Feeding,
            TBudgKenya$Feeding),Location=as.factor(c(rep("Males",nrow(TBudg))
            ,rep("Others",(nrow(TBudgHoffman)+nrow(TBudgKenya))))))
```

Kruskal Wallis test:

```r
kruskal.test(AllData$Feeding ~ AllData$Location)

##
##  Kruskal-Wallis rank sum test
##
## data:  AllData$Feeding by AllData$Location
## Kruskal-Wallis chi-squared = 13.5, df = 1, p-value = 0.0002386
```

⇨ Focal males forage less than baboons elsewhere.
